# Laying hen responses to multi-strain *Bacillus*-based probiotic supplementation from 25 to 37 weeks of age

**DOI:** 10.5713/ab.23.0495

**Published:** 2024-04-01

**Authors:** Elijah Ogola Oketch, Myunghwan Yu, Jun Seon Hong, Nuwan Chamara Chaturanga, Eunsoo Seo, Hans Lee, Rafael Gustavo Hermes, Natasja Smeets, Apichaya Taechavasonyoo, Susanne Kirwan, Raquel Rodriguez-Sanchez, Jung Min Heo

**Affiliations:** 1 Department of Animal Science and Biotechnology, Chungnam National University, Daejeon 34134, Korea; 2 Kemin Animal Nutrition and Health, Asia Pacific, 12 Senoko Drive, Singapore 758200; 3 Kemin Animal Nutrition and Health, Europa NV, Herentals 2200, Belgium

**Keywords:** Direct-fed Microbial, Egg Quality, Laying Hens, Microbiota, Productive Performance, Tibia

## Abstract

**Objective:**

This study aimed to investigate the efficacy of *Bacillus*-based probiotics supplemented at two different levels to modulate the productive performance, egg quality, tibia traits, and specific cecal bacteria counts of Hy-Line Brown layers from 25 to 37 weeks of age.

**Methods:**

A total of 216 twenty-five-week-old hens were randomly distributed into 3 experimental diets with 12 replicates of 6 birds per cage. Diets included basal diet supplemented with 0 (CON), 3×10^8^ (PRO1), or 3×10^9^ (PRO2) colony-forming unit (CFU) of the test probiotic containing *Bacillus subtilis* PB6, *Bacillus subtilis* FXA, and *Bacillus licheniformis* G3 per kilogram of feed.

**Results:**

Improved egg weights and mass at 29 weeks; and feed intake at 31 weeks (p<0.10) were noticed with the probiotic-supplemented PRO1 and PRO2 diets. Considering egg quality, the shell thickness, Haugh units, and yolk color were improved; but yolk cholesterol was lowered (p<0.05) with PRO1 and PRO2 diets at 29 weeks. At both 33 and 37 weeks, the egg-breaking strength, shell color and thickness, albumen height, Haugh units, and yolk color were improved; but yolk cholesterol was similarly lowered (p<0.05) with the PRO1 and PRO2 diets. Improved tibia Ca, ash, weights, and density; and raised cecal counts of *Bifidobacteria* and *Lactobacilli* (p<0.05) were noticed with PRO1 and PRO2 diets. Improved tibia P but reduced *Clostridia* counts (p<0.10) were also observed with the PRO1 and PRO2 diets.

**Conclusion:**

Probiotic supplementation of *Bacillus subtilis* PB6, *Bacillus subtilis* FXA, and *Bacillus licheniformis* G3 at 3×10^8^ CFU/kg of feed is adequate to significantly improve egg quality, lower yolk cholesterol, enhance several tibia traits, and raise the populations of beneficial cecal bacteria. Modest improvements in several productive parameters and tibia P but reduced *Clostridia* were also observed; and could warrant further investigation of probiotic effects beyond the current test period.

## INTRODUCTION

Antibiotic growth promoters (AGPs) have been perennially used in animal production to prevent or reduce diseases; and to improve performance. However, due to food safety concerns; and the transmission of antibiotic-resistant bacterial strains along the food chain, AGPs have since been banned in various jurisdictions [1]. The withdrawal of AGPs has negatively led to higher disease incidences and increased production costs. Recent research has therefore been focused on a variety of AGP alternatives collectively called nutraceuticals, with the potential to improve productive parameters and animal health [1]. Alongside other interventions, dietary probiotics have been investigated as one of the AGP alternatives.

Probiotics are non-pathogenic, live microbial feed supple ments that exert health and productive benefits to the host when supplied in adequate amounts. Several probiotic bacteria have been used including *Lactobacillus*, *Bacillus*, *Bifidobacterium*, *Streptococcus*, and *Enterococcus* [2,3]. Probiotic microbes should adhere to the epithelium, survive, and proliferate in the prevailing acidic environment in the gut; and remain viable under storage, processing, and transportation conditions [4,5]. Although the probiotic mode of action is complex and occurs by multiple pathways, it is suggested that through competitive exclusion and antagonism towards pathogenic bacteria, probiotics improve and maintain the host’s intestinal microbial balance thus, preventing dysbiosis [5,6]. Improved microbial diversity and balance is reported to enhance the colonization resistance against stressors; catalyze immune responses; promote the integrity of the gut architecture; and improve performance indices such as growth and laying rate for broilers and layers, respectively [2,5].

Considering probiotic supplementation for layers, several bacterial strains including *Lactobacillus*, *Enterococcus*, and *Saccharomyces cerevisiae* have been reported to result in the modulation of intestinal microbial populations, higher laying performance, and improved egg quality [7–9]. Suggesting probiotic involvement in mineral absorption and bone mineralization, increased tibia density, ash, and P contents have been reported with the supplemental *Bacillus* species [10,11]. Conversely, supplemental multi-strain probiotics containing *Bacillus subtilis* and *Bacillus licheniformis*, Mahdavi et al [12] reported no significant improvements in the laying performance and egg quality. The observed variabilities are attributed to several factors including, but not limited to, differences in the type of microbial species used, the dosage of administration, method of administration, environmental stress, and diet composition [13].

Due to the strain and/or species specificity of probiotics, and thus the variable responses upon supplementation, there is an ever-present need to evaluate novel probiotic products and other AGP alternatives for their effects on animal health and performance especially under the current requirements of AGP-free production. The current study examined the effect of supplementing different probiotic inclusion levels (3×10^8^ or 3×10^9^ colony-forming unit [CFU]/kg of feed) on: i) productive performance, ii) egg quality, iii) intestinal microbiota, and iv) tibia traits of laying hens. The test probiotic product is a mixed culture of *Bacillus subtilis* PB6, *Bacillus subtilis* FXA, and *Bacillus licheniformis* G3. It was reasoned that the multi-strain *Bacillus*-based probiotic would improve the productive performance and egg quality of layers. It was further expected that the probiotic would improve the populations of intestinal bacteria groups that are considered beneficial; and exert a positive influence on the mechanism behind bone mineralization and mineral absorption by improving the tibia traits. The possibility of incremental probiotic effects at higher inclusion levels was also examined.

## MATERIALS AND METHODS

The study was conducted at the Cheongyang Research Station of Chungnam National University. The experimental protocol and procedures for the current study were reviewed and approved by the Animal Ethics Committee of Chungnam National University (Protocol Number; 202206A-CNU-084). The test probiotic (Enterosure) was supplied by Kemin Animal Health. Enterosure is a mixed culture of Bacillus species including *Bacillus subtilis* PB6, *Bacillus subtilis* FXA, and *Bacillus licheniformis* G3.

### Birds, diets, and housing

In the current feeding trial, a total of two hundred and sixteen Hy-Line Brown layers were used. The 23-week-old birds were individually weighed upon arrival to verify the requirement of having approximately the same body weight. The hens were then randomly allocated to 36 cages with 12 replicate cages and 6 birds per cage; and taken through a two-week initial adaptation period to adjust and acclimatize to the surroundings. The birds were then equally fed one of the three dietary treatments, and eggs were collected daily to estimate the productive performance. Any deviations from the flock average were noticed, and birds that were not laying were excluded. Experimental diets included a basal diet with no probiotic that was formulated to meet the breed and age standards (CON); basal diet + probiotic at the level of 3×10^8^ CFU/kg of feed (PRO1); basal diet + probiotic at the level of 3×10^9^ CFU/kg of feed (PRO2), as shown in Table 1. The probiotic product was mixed into the basal diet as a powder to create the PRO1 and PRO2 diets.

A windowless and temperature-controlled (around 20°C to 22°C) facility was used to house the hens. The hens were subjected to a lighting scheme with 16 hours of continuous light and 8 hours of darkness. A total of 36 enriched cages measuring 90 cm high by 90 cm wide were used. Perches and nesting boxes were provided as enrichments in the cages to promote the welfare of the birds. Additionally, each cage was equipped with four nipple drinkers and a detachable feeder for the provision of water and feed.

### Productive performance

The total number and weight of eggs laid; and feed intake were recorded daily. The collected data was used to calculate the egg production and loss percentages, feed conversion ratios (FCRs), egg weight, and egg mass on a bi-weekly basis at the end of 27, 29, 31, 33, 35, and 37 weeks of age. The average egg production percentages on hen-day egg production basis (HDEP) were calculated as a function of the total number of eggs laid and the total number of hens per treatment. The egg loss percentages were calculated using the total number of spoilt/damaged eggs (dirty, rough, misshapen, cracked, shell-less) as a fraction of the total number of eggs produced. The FCR was calculated in terms of grams of total feed intake per day per hen divided by grams of total egg mass per hen per day. The egg mass was calculated as a factor of egg weight and hen-day egg production.

### Egg quality

At the end of 29, 33, and 37 weeks of age, a total of 36 eggs (3 eggs per replicate cage) were collected randomly and evaluated for egg quality. Eggshell breaking strength was evaluated using a texture analyzer (TA.XTplusC; Stable Micro Systems, Godalming, Surrey, UK). The shell color, albumen height, and Haugh units were measured using an egg multitester instrument (QCM+ Range; TSS, Dunnington, York, UK). Yolk color intensity was measured against the DSM yolk color fan (1, light yellow; 15, orange). A shell thickness micrometer (Digimatic MDC-MX Series; Mitutoyo, Aurora, IL, USA) was to measure the shell thickness at three different locations (upper, lower, and middle), excluding the inner shell membrane. Yolk cholesterol evaluation was conducted as per the procedure of Yalçın et al [14]. An egg yolk separator was used for separating the yolk and the albumen; their percentages relative to the total egg weight were determined. The internal egg quality and eggshell analyses were completed within 24 hours of egg collection.

### Tibia traits and specific cecal microbiota counts

At the end of the experiment (37 weeks of age), all birds were weighed on cage basis to determine the average final body weight. Subsequently, one bird per cage that was closer to the mean body weight (12 hens per treatment totaling 36 birds) was selected and sacrificed by carbon dioxide asphyxiation for the analysis of tibia traits and specific cecal microbiota counts. Following the procedure of Abdelqader et al [15], the left tibia was removed, de-fleshed, and dried for tibia analyses. Consequently, the tibiae weight in grams; and the tibiae volume using the displacement method was obtained. The method involved the dipping of de-fleshed tibia into a flask with an already recorded initial water volume. The difference between the initial water volume and the final water volume after dipping was recorded as the tibia volume. Subsequently, the tibia mass per unit volume (density) was then calculated from the weight and volume figures obtained. The tibiae were further analyzed for their total ash, Ca, and P.

For determining the counts of specific cecal microbiota, fresh cecal contents were obtained from the sacrificed hens and collected in sterile bags for further analysis. Collected samples were immediately diluted tenfold with sterile 0.9% NaCl and subsequently homogenized for 3 minutes as per the procedure of Abdelqader et al [15]. Bacterial counts were then performed using appropriate agar media. *Lactobacillus* spp., *Bifidobacterium* spp., *Clostridium* spp., and *Enterococcus* spp. were enumerated using Lactobacilli MRS agar, Beerens agar, reinforced clostridial agar, and Eosin-methylene-blue (EMB) lactose sucrose agar, respectively. *Lactobacillus*, *Bifidobacterium*, and *Clostridium* agar plates were incubated anaerobically for 48 hours at 39°C whereas EMB-agar plates were incubated for 24 hours at 37°C. The number of viable bacterial colonies was then counted immediately after removal from the incubator and expressed as log_10_ CFU/g of fresh cecal digesta sample.

### Statistical analyses

Collected data was analyzed using the general linear model procedure for the one-way analysis of variance echnique of IBM SPSS Statistics Windows, Version 26 (IBM Corp., Armonk, NY., USA). The cage was used as the experimental unit for assessing the productive performance and egg quality. Selected birds that were euthanized for sample collection were considered as the experimental unit for the tibia and specific cecal bacterial counts. Linear contrasts were examined to determine the response to supplemental probiotics. Statistical significance was measured at p<0.05, and trends (tendencies for significant effects) were measured at 0.05<p<0.10. Significant treatment effects were separated using Tukey’s multiple range test.

## RESULTS

### Productive performance

Improved egg weights and mass at the end of 29 weeks of age; and feed intake at the end of 31 weeks of age (p<0.10) were noticed with the probiotic-supplemented PRO1 and PRO2 diets, as detailed in Table 2.

### Internal egg and eggshell quality

At the end of 29 weeks of age, the recorded shell thickness, Haugh units, and yolk color were improved; but the yolk cholesterol was lowered (p<0.05) with the probiotic-supplemented PRO1 and PRO2 diets, as shown in Table 3. At the end of both 33 and 37 weeks, the egg-breaking strength, shell color and thickness, albumen height, Haugh units, and yolk color were improved; but yolk cholesterol was similarly lowered (p<0.05) with *Bacillus subtilis* PB6, *Bacillus subtilis* FXA, and *Bacillus licheniformis* G3 in the PRO1 and PRO2 diets.

### Tibia traits and specific cecal microbiota counts

Supplemental *Bacillus subtilis* PB6, *Bacillus subtilis* FXA, and *Bacillus licheniformis* G3 in the PRO1 and PRO2 diets improved (p<0.05) the tibia Ca, ash, weights, and density, as detailed in Table 4. Improved tibia P content (p<0.10) were also noticed with the probiotic-supplemented PRO1 and PRO2 relative to the non-supplemented CON diet. However, neither significant effects nor trends (0.05<p<0.10) were observed for the tibia length and volume that were measured.

Considering the cecal microbiota counts, higher *Bifidobacterium*, and *Lactobacillus* populations were observed (p< 0.05) with probiotics in the PRO1 and PRO2 diets, as reported in Table 4. Furthermore, reduced *Clostridium* populations (p<0.10) were noticed with supplemental probiotics in the PRO1 and PRO2 diets. Neither significant effects nor trends (0.05<p<0.10) were observed for the populations of *Enterococcus*.

## DISCUSSION

Research on potential AGP alternatives is essential to alleviate food safety concerns and the emergence of resistant bacterial strains with the use of AGPs in animal feeding. Additionally, the subsequent bans across various jurisdictions on the use of AGPs in animal nutrition have led to increased production costs and disease incidences. The use of probiotics as potential AGP alternatives is well-appreciated in literature and practice [1,2]. The safe use of probiotic bacteria is in the requirement to be innate to the gastrointestinal tract; hence the microbes can attach to the intestinal epithelium, survive, and proliferate under the prevailing acidic gut conditions [4,16]. Being innate to the gut, commonly-used probiotic bacteria are generally considered to be safe, non-pathogenic, and non-infective, even when supplemented at higher doses [16,17]. Probiotics have been reported to improve feed intake and utilization; stimulate immune response; and promote mucosal integrity [2,4,6]. These benefits contribute to improved gut health, i.e., the general presence of a stable and coordinated interaction between the diet, commensal microbiome, intestinal mucosa, and immune system in a symbiotic equilibrium that allows the gut to perform physiological functions, self-regulate, and withstand stressors [18]. Specifically, the efficacy of supplemental *Bacillus subtilis* PB6, *Bacillus subtilis* FXA, and *Bacillus licheniformis* G3 to increase the productive performance and egg quality; modulate the mechanism behind mineral absorption and bone mineralization; and improve the intestinal microbial balance of layers was investigated at two different levels.

Considering the productive performance, supplemental *Bacillus subtilis* PB6, *Bacillus subtilis* FXA, and *Bacillus licheniformis* G3 resulted in modest improvements in some measured parameters including egg weight, egg mass, and feed intake. Mahdavi et al [12] reported that a multi-strain probiotic containing *Bacillus subtilis* and *Bacillus licheniformis* did not improve parameters of productive performance including egg mass, weight, feed intake, and FCRs. Conversely, Ribeiro et al [19] and Abdelqader et al [11,15] reported the capacity of *Bacillus subtilis* to improve several productive performance metrics including egg production, egg mass, egg weight, and FCRs. The observed variabilities in the productive performance are not uncommon and are attributed to several factors including but not limited to, the differences in the type of microbial species used, the dosage of administration, diet composition, breed type, age of birds, and length of feeding [13]. Notably, the current values on productive performance parameters including HDEP and egg weights were relatively comparable to the expected standard values of the breed at the evaluated period of 25 to 37 weeks of age [20]. It is probable that with the increased focus on animal welfare using enriched cages at the appropriate stocking density; and the feeding of adequate diets, the hens were able to maintain the high productive performance that was recorded across the three experimental groups in the current study. The observation of modest probiotic-induced improvements in some parameters of productive performance could warrant further investigation beyond the current test period of 25 to 37 weeks of age into the late laying period.

Subject to improved nutrient utilization, the capacity of dietary probiotics to improve the internal egg and eggshell quality is well reported [8,9,21]. Concomitantly, the supplementation of *Bacillus subtilis* PB6, *Bacillus subtilis* FXA, and *Bacillus licheniformis* G3 in the current study improved the egg-breaking strength; yolk color intensity, and percentages; shell color and thickness; albumen height, and Haugh units. On the contrary, non-significant improvements in the internal egg and eggshell quality with probiotics containing several bacterial cultures have also been reported [22]. It is reasonable that the previously enumerated factors by Mikulski et al [13] contributed to the observed discrepancies. The observed discrepancies stress the species and/or strain specificity of probiotic bacteria and the need for constant evaluation of novel probiotic products. As a measure of albumen quality, Haugh units are a function of the albumen height and the egg weight. The observed improvements in the albumen heights and the resulting Haugh units point to the influence of direct fed microbials in increasing protein synthesis and water transfer from the yolk [21]. Notably, the recorded egg weights were not significantly improved with dietary probiotics. The inconsistency in terms of the significantly improved Haugh units, but unaffected egg weights with dietary probiotics could be explained by the high heritability of egg weights as a phenotypic trait [23]. Thus, it was not surprising that the egg weights were relatively comparable to the expected values of the breed and age [20].

Furthermore, improved egg-breaking strength is associated with thicker eggshells subject to supplemental *Bacillus subtilis* PB6, *Bacillus subtilis* FXA, and *Bacillus licheniformis* G3. As supported by previous studies using *Bacillus subtilis* cultures [15,24], the current improvements allude to a probiotic involvement in the absorption and utilization process of calcium and phosphorous to promote the overall eggshell quality. Apart from being a major determinant of consumer preference for table eggs, the darker eggshell colors that were noted in the current study are positively correlated to the improved breaking strength and eggshell thickness of the probiotic-supplemented birds; and could also point to improved photoantimicrobial defense against desiccation-resistant gram-positive bacteria as reported elsewhere [25]. Additionally, probiotic-induced improvements were noticed with much more concentrated egg yolk colors. These improvements could be attributed to a possible increase in the mobilization of lipid-soluble pigments including xanthophylls [26].

Suggesting further evidence of potential intervention in lipid metabolism, significant linear reductions in egg yolk cholesterol were observed with supplemental *Bacillus subtilis* PB6, *Bacillus subtilis* FXA, and *Bacillus licheniformis* G3. Previous studies have similarly reported the probiotic-induced lowering effect on yolk cholesterol [7,24]; and serum cholesterol [27,28]. Souza et al [29] reported lowered serum cholesterol levels using chromium propionate and a similar strain of *Bacillus subtilis* PB6 that was utilized in the current study. The probiotic-lowering effect on cholesterol is attributed to an improved internal environment for the proliferation of lactic acid bacteria (LAB); as supported by the current results on cecal bacterial counts showing improved *Lactobacillus* counts. It is reasonable that increased *Lactobacillus* populations exhibit an equally higher microbial bile salt deconjugating capacity that enhances the production of free bile salts through the action of bile salt hydrolase [5,27]. Free bile salts are known to co-precipitate cholesterol at lower pH values, and are less soluble in the small intestine thus, the salts are easily eliminated through fecal excretion [30]. Therefore, the elimination of cholesterol as a co-precipitate during the fecal excretion of bile salts reduces its availability for mobilization into the yolk. The excretion process additionally prevents bile salts from acting as precursors in cholesterol synthesis; more cholesterol will then be consequently directed towards de-novo bile acid synthesis, hence the lowered serum and yolk cholesterol levels that have been reported [27,31]. Additionally, it is plausible that enhanced LAB populations exhibit an equally improved assimilation capacity for dietary cholesterol for their own metabolism. These mechanisms could be responsible for reducing yolk cholesterol.

Due to a lowered efficiency of absorbing and depositing Ca in the eggshell with the increase in egg weights as laying hens age, observations of reduced eggshell quality during later stages of production as represented by higher egg loss percentages; and lowered eggshell weight and thickness have been reported [32–34]. Several interventions towards improved mineral absorption and bone mineralization have been investigated including dietary Ca supplementation [35]. However, dietary Ca supplementation could negatively impact the bioavailability of phosphorous, magnesium, and other trace elements that are known to influence eggshell quality [36]. Alternatively, a modulation of the mechanism behind mineral absorption and bone mineralization, that could result in higher calcium absorption improved eggshell quality [5,11]. In the current study, the capacity of dietary probiotics to enhance tibia characteristics including ash, P, Ca, weight, length, volume, and density was investigated. Supplemental *Bacillus subtilis* PB6, *Bacillus subtilis* FXA, and *Bacillus licheniformis* G3 significantly improved the tibia Ca, ash, weights, and density while marginally improving the analyzed tibia P content. Using several strains of *Bacillus* spp, Mutuş et al [10] and Abdelqader et al [11] similarly reported improved tibia traits including weight, density, and ash. The improved tibia traits are associated with enhanced bone mineralization, subject to higher calcium and phosphorous retention with probiotic feeding [5,7]. Using a multi-strain probiotic containing *Bacillus megaterium*, *Bacillus subtilis*, *Cupriavidus metallidurans*, and *Bacillus safensis*, Nkiambuo et al [37], observed improved eggshell Ca and P levels. It is reasonable that improved mineral retention resultantly improves the overall eggshell quality as corroborated by the current results on improved eggshell thickness, shell color, and egg-breaking strength.

There is considerable evidence pointing to the modula tion of the gut barrier function with direct fed microbials, resulting in improved mineral retention [38]. The hypothesis of improved gut barrier function is supported by the Lei et al [21] study that examined some indicative biomarkers of intestinal mucosa damage and injury including serum diamine oxidase (DAO) and _D_-lactate. They reported the capacity of supplemental *Bacillus licheniformis* to reduce _D_-lactate and serum DAO levels that are indicative of lowered intestinal injury and permeability; reduced gut barrier dysfunction; increased mucosal maturation; and enhanced membrane integrity [21,39]. Furthermore, probiotics may be also capable of digesting carbohydrates to produce metabolites including organic acids such as propionic, butyric, and acetic acids that have a lowering effect on the gut pH [5,31]. Lowered gut pH from the production of short-chain fatty acids and higher microbial populations (*Lactobacillus* and *Bifidobacterium*) creates a favorable acidic environment for the ionization of Ca and P, which is essential for mineral absorption [5,24]. These mechanisms might explain the previous reports of increased Ca and P retention with probiotics [7,37], as well as improved eggshell quality, higher tibiae Ca, and marginally improved P levels in the current study.

The role of the gut microbiota as an integral part of the gut health nexus alongside the diet, immune system, and intestinal mucosa, cannot be understated. Gut microbes engage in a variety of protective, structural, metabolic, and immune roles [40]. Therefore, several cecal microbiota populations were analyzed to assess the efficacy of improved microbial balance with *Bacillus subtilis* PB6, *Bacillus subtilis* FXA, and *Bacillus licheniformis* G3 supplementation. Significantly improved *Bifidobacterium* and *Lactobacillus* populations; as well as marginal reductions in *Clostridium* populations were observed with probiotic feeding. Though not significant, it is important to note the numerical reductions in the counts of enteric *Enterococcus* species in the probiotic-supplemented diets. The current results of improved cecal *Lactobacillus* and *Bifidobacterium* species agree with previous reports showing increased populations of beneficial bacteria and reduced counts of harmful bacteria with *Bacillus*-based probiotics [11]. Taken together, the improved levels of *Lactobacillus* and *Bifidobacterium* but reduced *Clostridium* and *Enterococcus* populations suggest the subtle manipulation of the intestinal environment for the desired probiotic colonization of beneficial intestinal microbiota through various mechanisms including, but not limited to, competitive exclusion [3,5]. Increased populations of favorable intestinal microbiota; and the constant communication that exists between intestinal epithelium, gut microbiota, and the immune system are responsible for maintaining mucosal integrity, barrier function, and overall gut health [4]. These interactions will ultimately facilitate the mobilization of nutrients for the improved tibia traits, and the internal egg and eggshell quality parameters that were observed in the current study.

## CONCLUSION

No further improvements were recorded with increasing probiotic supplementation at the rate of 3×10^9^ CFU/kg of feed. Supplemental *Bacillus subtilis* PB6, *Bacillus subtilis* FXA, and *Bacillus licheniformis* G3 at 3×10^8^ CFU/kg of feed is adequate to improve the internal egg and eggshell quality, lower yolk cholesterol, enhance several tibia traits; and raise the populations of some microbiota species that are considered beneficial. Reduced yolk cholesterol values due to supplemental probiotics might appeal to health-conscious consumers. Furthermore, enhanced tibia characteristics linked to the probiotic modulation of the mechanism behind mineral absorption and bone mineralization could translate into fewer damaged and cracked eggs as the hens age. Improved intestinal microbial balance through the colonization of beneficial intestinal microbiota is directly associated with the reported improvements in the internal egg and eggshell quality, and tibia characteristics of the supplemented hens. However, given that the probiotic was only fed till week 37 and probably as a result, marginal improvements were observed for the productive performance, the long-term effects of the tested probiotic beyond the current test period and into the late laying period should be examined.

## Figures and Tables

**Table 1:** Ingredients and calculated nutrient composition of the basal diet

Items	Amount, %
Ingredients	
Corn	58.37
Soybean meal, 44%	26.68
Beef tallow	2.92
Limestone	9.74
Mono-calcium phosphate	1.28
Iodized salt	0.50
DL-methionine	0.21
Vitamin-mineral premix^1)^	0.30
Calculated nutrient composition	
Dry matter	88.36
ME (kcal/kg)	2850
Crude protein	16.71
Crude fat	5.31
Crude fiber	3.27
SID Lysine	0.89
SID Methionine	0.44
SID Methionine + Cysteine	0.78
SID Threonine	0.53
SID Valine	0.67
Calcium	4.10
Total P	0.71
Available P	0.40

ME, metabolizable energy; SID, standardized ileal digestibility.

1) Provided per kilogram of diet: vitamin A (trans-retinyl acetate), 12,000 IU; vitamin D_3_ (cholecalciferol), 2,500 IU; vitamin E (DL-α-Tocopherol acetate), 30 IU; vitamin K_3_, 3 mg; D-pantothenic acid, 15 mg; nicotinic acid, 40 mg; choline, 400 mg; and vitamin B_12_, 12 μg; Fe (from iron sulfate), 90 mg; Cu (from copper sulfate), 8.8 mg; Zn (from zinc oxide), 100 mg; Mn (from manganese oxide), 54 mg; I (from potassium iodide), 0.35 mg; Se (from sodium selenite), 0.30 mg.

**Table 2:** Effects of probiotic *Bacillus subtilis* PB6, *Bacillus subtilis* FXA, and *Bacillus licheniformis* G3 supplementation on the productive performance of hens^1)^

Items	Diets^2)^			SEM	p-value^3)^

	CON	PRO1	PRO2		
wk 25–27					
Hen-day egg production (%)	93.25	94.25	94.44	1.406	n.s.
Feed intake (g/d/hen)	106.86	107.22	108.00	0.271	n.s.
FCR (g feed/g egg)	1.91	1.87	1.89	0.031	n.s.
Egg weight (g)	56.59	57.70	57.72	0.841	n.s.
Egg mass (g/d/hen)	52.81	54.24	54.38	1.027	n.s.
Egg loss percentage (%)	0.03	0.02	0.02	0.002	n.s.
wk 27–29					
Hen-day egg production (%)	94.05	95.04	94.84	1.317	n.s.
Feed intake (g/d/hen)	107.34	108.00	108.05	0.263	n.s.
FCR (g feed/g egg)	1.82	1.80	1.78	0.009	n.s.
Egg weight (g)	59.17	59.98	60.79	0.292	<0.10
Egg mass (g/d/hen)	55.61	56.97	57.60	0.753	<0.10
Egg loss percentage (%)	0.03	0.02	0.03	0.002	n.s.
wk 29–31					
Hen-day egg production (%)	94.35	95.93	95.44	0.954	n.s.
Feed intake (g/d/hen)	108.44	109.98	110.05	0.332	<0.10
FCR (g feed/g egg)	1.79	1.81	1.79	0.007	n.s.
Egg weight (g)	60.44	60.66	61.36	0.188	n.s.
Egg mass (g/d/hen)	57.04	58.20	58.53	0.597	n.s.
Egg loss percentage (%)	0.01	0.02	0.01	0.002	n.s.
Week 31–33					
Hen-day egg production (%)	93.85	94.54	94.05	0.777	n.s.
Feed intake (g/d/hen)	109.68	109.63	110.35	0.341	n.s.
FCR (g feed/g egg)	1.83	1.82	1.81	0.008	n.s.
Egg weight (g)	60.15	60.24	60.89	0.210	n.s.
Egg mass (g/d/hen)	56.45	56.94	57.27	0.494	n.s.
Egg loss percentage (%)	0.01	0.02	0.01	0.002	n.s.
wk 33–35					
Hen-day egg production (%)	92.86	95.24	93.95	0.826	n.s.
Feed intake (g/d/hen)	114.68	113.63	113.94	0.462	n.s.
FCR (g feed/g egg)	1.90	1.88	1.88	0.008	n.s.
Egg weight (g)	60.24	60.50	60.55	0.139	n.s.
Egg mass (g/d/hen)	55.93	57.61	56.86	0.481	n.s.
Egg loss percentage (%)	0.01	0.02	0.02	0.002	n.s.
wk 35–37					
Hen-day egg production (%)	90.58	93.65	91.27	0.865	n.s.
Feed intake (g/d/hen)	115.45	115.99	116.99	0.333	n.s.
FCR (g feed/g egg)	1.90	1.91	1.91	0.009	n.s.
Egg weight (g)	60.76	60.86	61.26	0.247	n.s.
Egg mass (g/d/hen)	54.99	57.01	55.88	0.522	n.s.
Egg loss percentage (%)	0.02	0.01	0.01	0.002	n.s.
wk 25–37					
Hen-day egg production (%)	93.15	94.78	94.00	0.318	n.s.
Feed intake (g/d/hen)	110.41	110.74	111.23	0.794	n.s.
FCR (g feed/g egg)	1.85	1.85	1.84	0.010	n.s.
Egg weight (g)	59.57	59.93	60.40	0.317	n.s.
Egg mass (g/d/hen)	55.48	56.81	56.77	0.349	n.s.
Egg loss percentage (%)	0.02	0.02	0.02	0.001	n.s.

SEM, pooled standard error of the mean; CFU, colony-forming unit; n.s., not significant.

1) Values are the mean of twelve replicates per treatment.

2) CON = 0 CFU/kg; PRO1 = 3×10^8^ CFU/kg; PRO2 = 3×10^9^ CFU/kg.

3) Statistical significance was determined at p<0.05 and trends (tendencies for significant effects) were measured at 0.05<p<0.10.

**Table 3:** Effects of probiotic *Bacillus subtilis* PB6, *Bacillus subtilis* FXA, and *Bacillus licheniformis* G3 supplementation on the internal egg and eggshell quality of hens^1)^

Items	Diets^2)^			SEM	p-value^3)^

	CON	PRO1	PRO2		
wk 29					
Egg-breaking strength (kg)	4.36	4.46	4.54	1.008	n.s.
Shell color (%)	25.90	26.66	26.80	0.404	n.s.
Shell thickness (mm)	0.35^a^	0.37^b^	0.35^a^	0.003	<0.05
Albumen height (mm)	8.12	8.36	8.40	0.142	n.s.
Haugh units	89.46^a^	95.92^b^	96.13^b^	0.691	<0.05
Yolk color	7.60^a^	8.43^b^	8.40^b^	0.113	<0.05
Yolk cholesterol	15.72^b^	10.11^a^	10.01^a^	0.537	<0.05
Yolk percentage	25.55	25.64	26.13	0.246	n.s.
Albumen percentage	60.78	61.04	60.54	0.340	n.s.
wk 33					
Egg-breaking strength (kg)	4.23^a^	4.71^b^	4.65^ab^	0.843	<0.05
Shell color (%)	25.20^a^	27.18^b^	27.27^b^	0.349	<0.05
Shell thickness (mm)	0.33^a^	0.37^b^	0.36^b^	0.004	<0.05
Albumen height (mm)	8.08^a^	8.60^b^	8.62^b^	0.074	<0.05
Haugh units	95.14^a^	99.86^b^	99.99^b^	0.441	<0.05
Yolk color	7.33^a^	8.23^b^	8.70^b^	0.112	<0.05
Yolk cholesterol	17.76^b^	13.15^a^	13.01^a^	0.755	<0.05
Yolk percentage	24.68	25.37	24.76	0.243	n.s.
Albumen percentage	61.31	61.20	61.67	0.432	n.s.
wk 37					
Egg-breaking strength (kg)	4.25^a^	4.75^b^	4.90^b^	0.851	<0.05
Shell color (%)	26.07^a^	28.83^b^	28.33^b^	0.461	<0.05
Shell thickness (mm)	0.32^a^	0.37^b^	0.37^b^	0.003	<0.05
Albumen height (mm)	8.10^a^	8.62^b^	8.60^b^	0.096	<0.05
Haugh units	95.73^a^	99.08^b^	99.57^b^	0.394	<0.05
Yolk color	7.70^a^	8.36^b^	8.33^b^	0.099	<0.05
Yolk cholesterol	13.65^b^	9.81^a^	9.72^a^	0.481	<0.05
Yolk percentage	25.88	26.31	26.41	0.238	n.s.
Albumen percentage	60.75	59.64	60.16	0.365	n.s.

SEM, pooled standard error of the mean; CFU, colony-forming unit; n.s., not significant.

1) Values are the mean of 12 replicates per treatment.

2) CON = 0 CFU/kg; PRO1= 3×10^8^ CFU/kg; PRO2 = 3×10^9^ CFU/kg.

3) Statistical significance was determined at p<0.05 and trends (tendencies for significant effects) were measured at 0.05<p<0.10.

a,b Means with different superscripts within the same column differ significantly (p<0.05).

**Table 4:** Effects of probiotic *Bacillus subtilis* PB6, *Bacillus subtilis* FXA, and *Bacillus licheniformis* G3 supplementation on tibia traits and specific cecal microbiota counts in log_10_ CFU/g of fresh cecal digesta^1)^

Items	Diets^2)^			SEM	p-value^3)^

	CON	PRO1	PRO2		
Tibia traits					
Tibia weight (g)	13.00^a^	15.33^b^	15.67^b^	0.370	<0.05
Tibia length (cm)	12.26	12.38	12.43	0.085	n.s.
Tibia volume (cm^3^)	9.50	9.67	9.50	0.166	n.s.
Tibia density (g/cm^3^)	1.37^a^	1.59^b^	1.66^b^	0.045	<0.05
Tibia P (%)	14.10	15.25	15.34	0.245	<0.10
Tibia Ca (%)	39.72^a^	42.52^b^	42.47^b^	0.539	<0.05
Tibia ash (%)	51.40^a^	55.95^b^	56.18^b^	0.834	<0.05
Specific cecal microbiota counts					
*Lactobacillus*	5.88^a^	6.57^b^	6.58^b^	0.129	<0.05
*Bifidobacterium*	5.72^a^	6.31^b^	6.63^b^	0.130	<0.05
*Enterococcus*	4.75	4.63	4.61	0.146	n.s.
*Clostridium*	5.93	5.18	5.27	0.152	<0.10

CFU, colony-forming unit; SEM, pooled standard error of the mean; n.s., not significant.

1) Values are the mean of 12 replicates per treatment.

2) CON = 0 CFU/kg; PRO1 = 3 ×10^8^ CFU/kg; PRO2 = 3 ×10^9^ CFU/kg.

3) Statistical significance was determined at p<0.05 and trends (tendencies for significant effects) were measured at 0.05<p<0.10.

a,b Means with different superscripts within the same column differ significantly (p<0.05).
